# Case Report: Toripalimab Combined With Anlotinib in a Patient With Metastatic Upper Tract Urothelial Carcinoma After Pembrolizumab Failure

**DOI:** 10.3389/fonc.2022.796407

**Published:** 2022-02-28

**Authors:** Ning Zan, Xuan Zhang, Lingyan Du, Zhiyu Lin, Danfei Yu, Juan Liu, Fusheng Gou

**Affiliations:** Department of Oncology and Hematology, People’s Hospital of Leshan, Leshan, China

**Keywords:** upper tract urothelial carcinoma, immunotherapy, antiangiogenesis therapy, toripalimab, anlotinib, immune checkpoint inhibitor, PD-1

## Abstract

Urothelial carcinoma is the most common primary upper tract urinary carcinoma. If surgery, chemotherapy, and immunotherapy fail, the prognosis for upper tract urinary carcinoma is extremely poor. Immunotherapy combined with antiangiogenesis therapy is a new therapeutic regimen with a synergistic antitumor effect. We present a case of metastatic upper tract urinary carcinoma in which the patient underwent surgery and treatment with gemcitabine combined with platinum-based chemotherapy. Radiotherapy and second-line immunotherapy (pembrolizumab) were administered after the cancer had progressed to the left lymph node of the abdominal aorta in the umbilical plane. However, the patient developed liver metastases while being treated with pembrolizumab. He was administered off-label immunotherapy (toripalimab) combined with antiangiogenesis therapy (anlotinib) and achieved a long-term clinical response for over 25 months. Toripalimab combined with anlotinib has potential therapeutic value for locally advanced or metastatic upper tract urinary carcinoma in patients who had previously received platinum-based chemotherapy and had disease progression or after treatment with a PD-1 inhibitor.

## Introduction

Urothelial carcinoma is the most common type of primary upper tract urinary carcinoma (UTUC). The first option for UTUC is surgery. For advanced and metastatic UTUC, platinum-based chemotherapy is the preferred treatment. However, the median overall survival (OS) is only 12.5–15.5 months, and almost all patients experience disease progression (1). Developing new treatment strategies is crucial, especially for advanced and metastatic UTUC. Immunotherapy, particularly pembrolizumab, is the main option for second-line treatment following platinum-based chemotherapy. It increased OS by 2.9 months compared to standard paclitaxel, docetaxel, or vinflunine in a randomized, phase III trial (2). A positive objective response rate was also observed in clinical trials involving nivolumab (3), avelumab (4), and atezolizumab (5). Moreover, avelumab administered as maintenance therapy after a platinum-based first-line treatment increased the median OS by 7.1 months compared to the supportive care for advanced or metastatic urothelial carcinoma (6). Once immunotherapy fails, patients who had received platinum-containing chemotherapy and immunotherapy can choose enfortumab vedotin for locally advanced or metastatic urothelial carcinoma (7) or erdafitinib for locally advanced, unresectable, or metastatic urothelial carcinoma with fibroblast growth factor receptor (FGFR) alterations (8). However, there are no definite guidelines for recommending combination immunotherapy regimens after failure of second-line immunotherapy. Using a combination of immunotherapy and antiangiogenic therapy to treat UTUC after second-line immunotherapy failure has not been reported, although this approach has been used in other cancers. For instance, nivolumab combined with cabozantinib is used to treat renal cell carcinoma (9). Cabozantinib also has an immunomodulatory effect in relapsed/refractory metastatic urothelial carcinoma (10). This provides a rationale for combining antiangiogenic and immunotherapeutic treatments. This report presents a case of metastatic UTUC that achieved long-term clinical response after pembrolizumab failure when treated with toripalimab and anlotinib.

## Case Description

**Figure 1:**
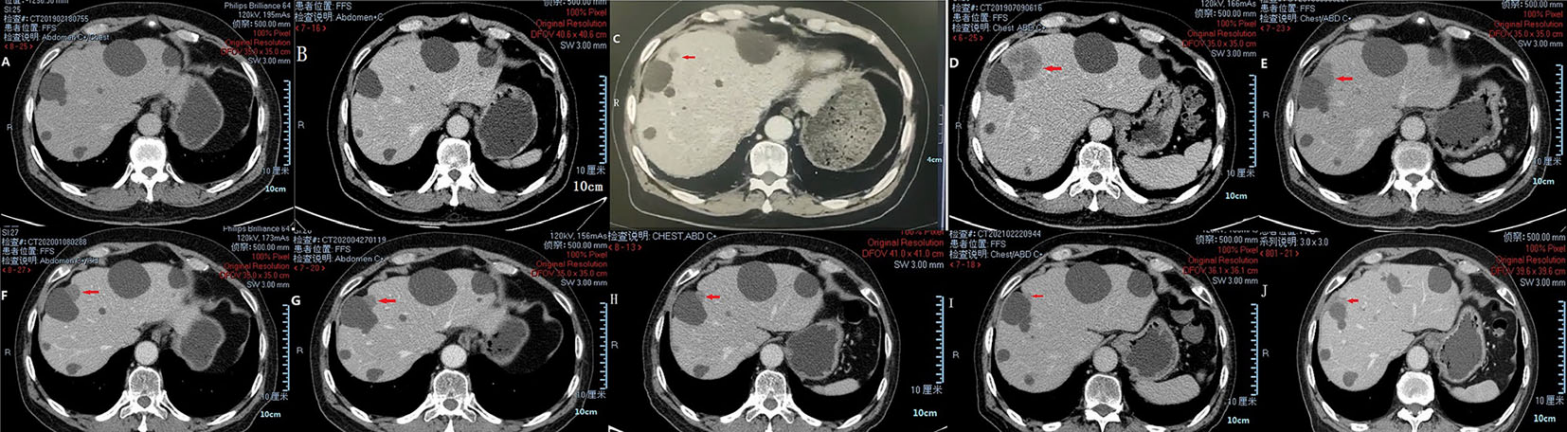
CT images of liver metastases during treatment of pembrolizumab and toripalimab combined with anlotinib. Multiple liver cysts **(A, B)**. Multiple new lesions in the liver **(C)**. Enlarged new liver lesions **(D)**. Liver lesions controlled *via* treatment **(E–J)**.

**Figure 2:**
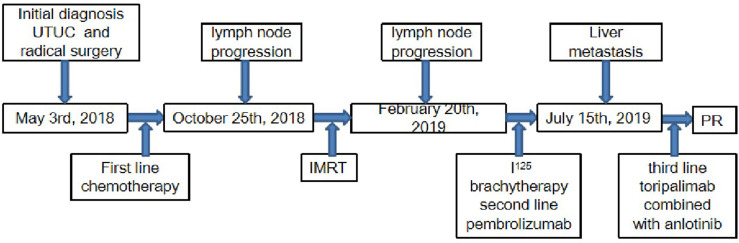
Timeline of the patient’s treatment.

In March 2018, a 71-year-old Chinese male was initially admitted to the West China Hospital of Sichuan University for hypogastralgia, which had lasted 2 months, and remained hospitalized. The patient had no family history of cancer. Computed tomography (CT) scans revealed the possibility of ureteral carcinoma. The lumen of some segments of the left ureter was inhomogeneously dilated. The lumen of multiple segments could not be visualized. Multiple soft tissue density nodules and masses with a large cross-section of about 3.5 × 2.1 cm were observed. The adjacent fat space was blurred. Peripheral lymph nodes were increased and enlarged. The left renal margin and renal pelvis wall were rough. Nodules were seen in the left adrenal gland. The patient underwent a ureteroscopy under general anesthesia on April 24, 2018. The ureteroscope revealed a yellowish-white flocculent neoplasm with a diameter of 4 cm in the left ureter. The surgeon took three specimens using biopsy forceps for examination. Histopathology indicated that the left ureter neoplasm was fibrous tissue hyperplasia with inflammatory cell infiltration. However, a few heterologous cells were found in the superficial mucosa. Immunohistochemical results indicated a high suspicion of urothelial carcinoma, but only a few idioblasts were found in the tissue. The immunohistochemical staining results were as follows: GATA-3 (+), P63 (+), P53 (+), CD44 (+), CK20 (−), and Ki-67 (+30%). The patient agreed to undergo exploratory surgery to accurately identify the pathology type and receive radical surgery if the surgeon found it possible. On May 3, 2018, the patient underwent surgery, and during exploratory surgery, the surgeon found that a radical operation could be performed. The patient received a radical resection of the left ureteral carcinoma. The surgeon observed that the left ureter had thickened, and the ureteral lumen (with a diameter of 2–4 cm) had disappeared. The lymph nodes were diffusely enlarged and partially fused next to the left common iliac artery, iliac artery bifurcation, and external iliac artery. A lesion (with a volume of 4 × 3 × 2 cm) in the descending mesocolon near the left renal artery level was found. The postoperative histopathological findings revealed a high-grade invasive urothelial carcinoma with adenoid differentiation and squamous metaplasia. The tumor had also invaded surrounding tissues, including the periureteral adipose tissue, perirenal adipose tissue, and renal parenchyma. A lymph node metastasis at the iliac artery bifurcation and a cancerous nodule in a mesenteric lesion was found. Immunohistochemical staining results were as follows: GATA-3 (+), CK5/6 (+), P63 (+), CK7 (+), CK20 (−), CgA (−), Syn (−), and PDL1 (+; about 70%). The patient was diagnosed with UTUC (stage IV, T4N1M1) based on disease history, symptoms, and examination findings. Beginning in May 2018, the patient was treated with gemcitabine (1000 mg/m^2^, intravenous [IV], days 1 and 8) and cisplatin (75 mg/m^2^, IV, day 1) every 3 weeks for one cycle. He continued to be treated with gemcitabine (1000 mg/m^2^, IV, days 1 and 8) and nedaplatin (80 mg/m^2^, IV, day 1) every 3 weeks for one cycle due to a decrease in creatinine clearance. On June 29, 2018, abdominal contrast-enhanced CT revealed a suspiciously thickened inner segment of the ureter bladder wall, a slightly enlarged left lymph node of the abdominal aorta in the umbilical plane was, and a thickened bladder wall. The patient was treated with gemcitabine (1000 mg/m^2^, IV, days 1 and 8) and nedaplatin (80 mg/m^2^, IV, day 1) every 3 weeks for one cycle. On July 17, 2018, a right ureteroscopy revealed that the right ureter and bladder were normal. The patient continued to be treated with gemcitabine (1000 mg/m^2^, IV, days 1 and 8) and nedaplatin (80 mg/m^2^, IV, day 1) every 3 weeks for two cycles. On October 25, 2018, abdominal contrast-enhanced CT revealed progressive disease (PD) in the left lymph node of the abdominal aorta in the umbilical plane, according to the response evaluation criteria in solid tumors 1.1 (RECIST1.1). Because there was only one isolated lesion, the patient received intensity-modulated radiation therapy for the lymph node. On December 13, 2018, abdominal contrast-enhanced CT revealed that the lymph node was slightly enlarged. The effective evaluation was stable disease (SD) according to the RECIST1.1. On February 20, 2019, abdominal contrast-enhanced CT revealed PD in the left lymph node and multiple liver cysts (**Figure 1A**). Compared with the cysts observed on June 29, 2018, the liver cysts persisted and did not change (**Figure 1B**). The patient received I^125^ interstitial brachytherapy to control the progression of the lymph node on March 21, 2019, and was treated with pembrolizumab (200 mg, IV, day 1) every 3 weeks starting on April 6, 2019. The patient had no other obvious adverse drug reactions. On May 13, 2019, contrast-enhanced CT revealed multiple new lesions in the liver (**Figure 1C**). The patient refused positron emission tomography/computed tomography (PET/CT). The patient was treated with pembrolizumab because the imaging features of the new liver lesions were not typical for tumors. As of July 15, 2019, contrast-enhanced CT revealed the disappearance of the lymph node. Multiple new lesions appeared in the liver (**Figure 1D**). The imaging features of the new liver lesions were enlarged and typical for tumors. The effective evaluation of the liver lesions was PD according to the RECIST1.1. The patient was administered off-label toripalimab and anlotinib with his consent. The patient was treated with toripalimab (240 mg, IV, day 1) and anlotinib (12 mg, oral, days 1–14) every 3 weeks beginning July 23, 2019. He developed lower limb weakness after the first cycle of toripalimab combined with anlotinib [Common Terminology Criteria for Adverse Events (CTCAE) grade 1]. Symptoms improved after rest. On September 2, 2019, the patient developed herpes zoster after the second cycle of toripalimab combined with anlotinib (CTCAE grade 2), which improved after 2 weeks of treatment with valacyclovir hydrochloride tablets and aciclovir cream. The patient continued to be treated with toripalimab and anlotinib as per the recommended dosage. On October 8, 2019, contrast-enhanced CT revealed significantly reduced liver lesions (**Figure 1E**). The effective evaluation was “partial response”, according to RECIST1.1. The patient continued to be treated with toripalimab (240 mg, IV, day 1) and anlotinib (12 mg, oral, day 1–day 14) every 3 weeks. Contrast-enhanced CT revealed that the metastatic liver lesions achieved long-term SD according to RECIST1.1 as of January 10, 2020 (**Figure 1F**), May 8, 2020 (**Figure 1G**), September 21, 2020 (**Figure 1H**), February 25, 2021 (**Figure 1I**), and June 30, 2021 (**Figure 1J**). The patient continued treatment with toripalimab (240 mg, IV, day 1) and anlotinib (12 mg, oral, day 1–day 14) every 3 weeks, and the disease has been under control for over 25 months. The timeline of the patient’s treatment is shown in **Figure 2**.

## Discussion

In recent years, studies have confirmed that immune checkpoint inhibitors (ICIs) show positive efficacy in both second-line and first-line treatments for advanced urothelial carcinoma (11, 12). Although the effect of ICIs is stable and lasting, drug resistance will occur. There are no clear guidelines or recommendations for combination immunotherapy after second-line immunotherapy fails.

Immunotherapy combined with antiangiogenic therapy has a synergistic effect in antitumor therapy (13). The antitumor effects of antiangiogenic therapy and immunotherapy are closely related to the tumor microenvironment (14). Immunotherapy is most effective when the inflammatory response is activated in the tumor microenvironment (15). Antiangiogenic drugs can increase the infiltration of lymphocytes into tumors and further reverse the immunosuppressive state of the tumor microenvironment, improving the efficacy of ICIs (16), thereby inhibiting the formation of tumor blood vessels and normalizing the vasculature surrounding the tumor. Vascular normalization improves the antitumor immune response (17) and enhances the initiation and activation of T cells in the presentation of tumor antigen. Tissue hypoperfusion causes immunosuppressive cells to gather in the hypoxic environment and inhibits the activation of immune cells (18–22). Vascular normalization also enhances the tissue perfusion and T cell infiltration of the tumor and formation of an inflammatory immune environment.

The IMpower150 clinical trial demonstrated the synergistic effect of immunotherapy combined with antiangiogenic therapy (13). The combination of pembrolizumab with ramucirumab showed a favorable manageable safety and antitumor activity in patients with urothelial carcinoma (23). Several preclinical experiments and phase I, II, and III clinical trials have shown the antitumor efficacy of immunotherapy combined with antiangiogenic therapy in treating urogenital tumors (24). Pembrolizumab plus lenvatinib therapy in ICI-pretreated patients with renal cell carcinoma showed an objective response rate of 55.8% at 24 weeks; the combination therapy demonstrated positive antitumor activity and manageable safety (25). This result provides a rationale for combining antiangiogenic and immunotherapeutic treatments after immunotherapy failure.

In this case, the patient developed liver metastases during pembrolizumab treatment. He continued with toripalimab and anlotinib treatment and has achieved a long-term clinical response. Toripalimab is a recombinant, humanized PD-1 monoclonal antibody that is well tolerated and has demonstrated promising antitumor activity in urologic cancers (26). Anlotinib is a small-molecule tyrosine kinase inhibitor. Its targets include vascular endothelial growth factor receptors 1, 2, and 3; fibroblast growth factor receptors (FGFR1, FGFR2, FGFR3, and FGFR4); c-Kit; and platelet-derived growth factor receptors α and β. Furthermore, it can also inhibit tumor angiogenesis and tumor cell proliferation (27–29). FGFR2/3 mutation and fusion are common problems in urothelial carcinoma patients (30). About 20% of advanced urothelial carcinoma patients and up to 37% of UTUC patients have FGFR mutations (31, 32). FGFRs are also targeted by anlotinib. Unfortunately, the FGFR status of our patient is not known.

In this case, the patient achieved a long-term clinical response when treated with toripalimab combined with anlotinib after pembrolizumab failure. This finding has potential therapeutic value for locally advanced or metastatic UTUC in patients who had previously received platinum-containing chemotherapy and had had disease progression during or after treatment with a PD-1 inhibitor. However, additional studies and clinical trials are needed to establish the value of this approach.

## Data Availability Statement

The datasets for this study can be found in the supplementary material. Further inquiries can be directed to the corresponding author.

## Ethics Statement

Written informed consent was obtained from the individual(s) for the publication of any potentially identifiable images or data included in this article.

## Author Contributions

NZ wrote the paper. XZ collected the case data. LD collected the information. ZL guided article writing. DY prepared the photos. JL and FG proofread the manuscript. All authors contributed to the article and approved the submitted version.

## Conflict of Interest

The authors declare that the research was conducted in the absence of any commercial or financial relationships that could be construed as a potential conflict of interest.

## Publisher’s Note

All claims expressed in this article are solely those of the authors and do not necessarily represent those of their affiliated organizations, or those of the publisher, the editors and the reviewers. Any product that may be evaluated in this article, or claim that may be made by its manufacturer, is not guaranteed or endorsed by the publisher.

## Abbreviations

CT, computed tomography; CTCAE, Common Terminology Criteria for Adverse Events; FGFR, fibroblast growth factor receptor; OS, overall survival; PD, progressive disease; RECIST, response evaluation criteria in solid tumors; SD, stable disease; UTUC, upper tract urinary carcinoma; ICI, immune checkpoint inhibitor; IV, intravenous.

## References

[1] Bellmunt J Fougeray R Rosenberg JE von der Maase H Schutz FA Salhi Y. Long-Term Survival Results of a Randomized Phase III Trial of Vinflunine Plus Best Supportive Care Versus Best Supportive Care Alone in Advanced Urothelial Carcinoma Patients After Failure of Platinum-Based Chemotherapy. Ann Oncol (2013) 24(6):1466–72. doi: 10.1093/annonc/mdt007 23419284

[2] Bellmunt J de Wit R Vaughn DJ Fradet Y Lee JL Fong L. Pembrolizumab as Second-Line Therapy for Advanced Urothelial Carcinoma. N Engl J Med (2017) 376(11):1015–26. doi: 10.1056/NEJMoa1613683 PMC5635424 28212060

[3] Sharma P Retz M Siefker-Radtke A Baron A Necchi A Bedke J. Nivolumab in Metastatic Urothelial Carcinoma After Platinum Therapy (CheckMate 275): A Multicentre, Single-Arm, Phase 2 Trial. Lancet Oncol (2017) 18(3):312–22. doi: 10.1016/S1470-2045(17)30065-7 28131785

[4] Patel MR Ellerton J Infante JR Agrawal M Gordon M Aljumaily R. Avelumab in Metastatic Urothelial Carcinoma After Platinum Failure (JAVELIN Solid Tumor): Pooled Results From Two Expansion Cohorts of an Open-Label, Phase 1 Trial. Lancet Oncol (2018) 19(1):51–64. doi: 10.1016/S1470-2045(17)30900-2 29217288 PMC7984727

[5] Rosenberg JE Hoffman-Censits J Powles T van der Heijden MS Balar AV Necchi A. Atezolizumab in Patients With Locally Advanced and Metastatic Urothelial Carcinoma Who Have Progressed Following Treatment With Platinum-Based Chemotherapy: A Single-Arm, Multicentre, Phase 2 Trial. Lancet (2016) 387(10031):1909–20. doi: 10.1016/S0140-6736(16)00561-4 PMC5480242 26952546

[6] Powles T Park SH Voog E Caserta C Valderrama BP Gurney H. Avelumab Maintenance Therapy for Advanced or Metastatic Urothelial Carcinoma. N Engl J Med (2020) 383(13):1218–30. doi: 10.1056/NEJMoa2002788 32945632

[7] Powles T Rosenberg JE Sonpavde GP Loriot Y Durán I Lee JL. Enfortumab Vedotin in Previously Treated Advanced Urothelial Carcinoma. N Engl J Med (2021) 384(12):1125–35. doi: 10.1056/NEJMoa2035807 PMC8450892 33577729

[8] Loriot Y Necchi A Park SH Garcia-Donas J Huddart R Burgess E. Erdafitinib in Locally Advanced or Metastatic Urothelial Carcinoma. N Engl J Med (2019) 381(4):338–48. doi: 10.1056/NEJMoa1817323 31340094

[9] Choueiri TK Powles T Burotto M Escudier B Bourlon MT Zurawski B. Nivolumab Plus Cabozantinib Versus Sunitinib for Advanced Renal-Cell Carcinoma. N Engl J Med (2021) 384(9):829–41. doi: 10.1056/NEJMoa2026982 PMC8436591 33657295

[10] Apolo AB Nadal R Tomita Y Davarpanah NN Cordes LM Steinberg SM. Cabozantinib in Patients With Platinum-Refractory Metastatic Urothelial Carcinoma: An Open-Label, Single-Centre, Phase 2 Trial. Lancet Oncol (2020) 21(8):1099–109. doi: 10.1016/S1470-2045(20)30202-3 PMC8236112 32645282

[11] Donin NM Lenis AT Holden S Drakaki A Pantuck A Belldegrun A. Immunotherapy for the Treatment of Urothelial Carcinoma. J Urol (2017) 197(1):14–22. doi: 10.1016/j.juro.2016.02.3005 27460757

[12] Gupta S Gill D Poole A Agarwal N. Systemic Immunotherapy for Urothelial Cancer: Current Trends and Future Directions. Cancers (2017) 9(2):15. doi: 10.3390/cancers9020015 PMC5332938 28134806

[13] Reck M Mok TSK Nishio M Jotte RM Cappuzzo F Orlandi F. Atezolizumab Plus Bevacizumab and Chemotherapy in Non– Small- Cell Lung Cancer (IMpower150): Key Subgroup Analyses of Patients With EGFR Mutations or Baseline Liver Metastases in a Randomised, Open- Label Phase 3 Trial. Lancet Respir Med (2019) 7(5):387–401. doi: 10.1016/S2213-2600(19)30084-0 30922878

[14] De Palma M Biziato D Petrova TV. Microenvironmental Regulation of Tumour Angiogenesis. Nat Rev Cancer (2017) 17(8):457–74. doi: 10.1038/nrc.2017.51 28706266

[15] Hegde PS Karanikas V Evers S. The Where, the When, and the How of Immune Monitoring for Cancer Immunotherapies in the Era of Checkpoint Inhibition. Clin Cancer Res (2016) 22(8):1865–74. doi: 10.1158/1078-0432.CCR-15-1507 27084740

[16] Oelkrug C Ramage JM. Enhancement of T Cell Recruitment and Infiltration Into Tumours. Clin Exp Immunol (2014) 178(1):1–8. doi: 10.1111/cei.12382 PMC4360188 24828133

[17] Huang Y Goel S Duda DG Fukumura D Jain RK. Vascular Normalization as an Emerging Strategy to Enhance Cancer Immunotherapy. Cancer Res (2013) 73(10):2943–8. doi: 10.1158/0008-5472.CAN-12-4354 PMC3655127 23440426

[18] Ramjiawan RR Griffioen AW Duda DG. Antiangiogenesis for Cancer Revisited: Is There a Role for Combinations With Immunotherapy? Angiogenesis (2017) 20(2):185–204. doi: 10.1007/s10456-017-9552-y 28361267 PMC5439974

[19] Viallard C Larrivée B. Tumor Angiogenesis and Vascular Normalization: Alternative Therapeutic Targets. Angiogenesis (2017) 20(4):409–26. doi: 10.1007/s10456-017-9562-9 28660302

[20] Pircher A Wolf D Heidenreich A Hilbe W Pichler R Heidegger I. Synergies of Targeting Tumor Angiogenesis and Immune Checkpoints in Non-Small Cell Lung Cancer and Renal Cell Cancer: From Basic Concepts to Clinical Reality. Int J Mol Sci (2017) 18(11):2291. doi: 10.3390/ijms18112291 PMC5713261 29088109

[21] Robert L Ribas A Hu-Lieskovan S. Combining Targeted Therapy With Immunotherapy. Can 1 + 1 Equal More Than 2? Semin Immunol (2016) 28(1):73–80. doi: 10.1016/j.smim.2016.01.001 26861544 PMC4933650

[22] Allen E Jabouille A Rivera LB Lodewijckx I Missiaen R Steri V. Combined Antiangiogenic and Anti-PD-L1 Therapy Stimulates Tumor Immunity Through HEV Formation. Sci Transl Med (2017) 9(385):eaak9679. doi: 10.1126/scitranslmed.aak9679 PMC5554432 28404866

[23] Herbst RS Arkenau H-T Santana-Davila R Calvo E Paz-Ares L Cassier PA. Ramucirumab Plus Pembrolizumab in Patients With Previously Treated Advanced Non-Small-Cell Lung Cancer, Gastrooesophageal Cancer, or Urothelial Carcinomas (JVDF): A Multicohort, non-Randomised, Open-Label, Phase 1a/B Trial. Lancet Oncol (2019) 20(8):1109–23. doi: 10.1016/S1470-2045(19)30458-9 31301962

[24] Zhu N Weng S Wang J Chen J Yu L Fang X. Preclinical Rationale and Clinical Efficacy of Antiangiogenic Therapy and Immune Checkpoint Blockade Combination Therapy in Urogenital Tumors. J Cancer Res Clin Oncol (2019) 145(12):3021–36. doi: 10.1007/s00432-019-03044-5 PMC11810195 31617075

[25] Lee CH Shah AY Rasco D Rao A Taylor MH Di Simone C. Lenvatinib Plus Pembrolizumab in Patients With Either Treatment-Naive or Previously Treated Metastatic Renal Cell Carcinoma (Study 111/KEYNOTE-146): A Phase 1b/2 Study. Lancet Oncol (2021) 22(7):946–58. doi: 10.1016/S1470-2045(21)00241-2 PMC8316679 34143969

[26] Tang B Yan X Sheng X Si L Cui C Kong Y. Safety and Clinical Activity With an Anti-PD-1 Antibody JS001 in Advanced Melanoma or Urologic Cancer Patients. J Hematol Oncol (2019) 12(1):7. doi: 10.1186/s13045-018-0693-2 30642373 PMC6332582

[27] Shen G Zheng F Ren D Du F Dong Q Wang Z. Anlotinib: A Novel Multi-Targeting Tyrosine Kinase Inhibitor in Clinical Development. J Hematol Oncol (2018) 11(1):120. doi: 10.1186/s13045-018-0664-7 30231931 PMC6146601

[28] Xie C Wan X Quan H Zheng M Fu L Li Y. Preclinical Characterization of Anlotinib, a Highly Potent and Selective Vascular Endothelial Growth Factor Receptor-2 Inhibitor. Cancer Sci (2018) 109(4):1207–19. doi: 10.1111/cas.13536 PMC5891194 29446853

[29] Taurin S Yang CH Reyes M Cho S Coombs DM Jarboe EA. Abstract 3244: Treatment of Endometrial Cancer Cells With a New Small Tyrosine Kinase Inhibitor Targeting Mutated Fibroblast Growth Factor Receptor-2. Cancer Res (2017) 77(13_Supplement):3244. doi: 10.1158/1538-7445.AM2017-3244 28428276

[30] Haugsten EM Wiedlocha A Olsnes S Wesche J. Roles of Fibroblast Growth Factor Receptors in Carcinogenesis. Mol Cancer Res (2010) 8(11):1439–52. doi: 10.1158/1541-7786.MCR-10-0168 21047773

[31] Knowles MA Hurst CD. Molecular Biology of Bladder Cancer: New Insights Into Pathogenesis and Clinical Diversity. Nat Rev Cancer (2015) 15(1):25–41. doi: 10.1038/nrc3817 25533674

[32] Li Q Bagrodia A Cha EK Coleman JA. Prognostic Genetic Signatures in Upper Tract Urothelial Carcinoma. Curr Urol Rep (2016) 17(2):12. doi: 10.1007/s11934-015-0566-y 26757906 PMC4823991

